# The Practical Anatomist

**Published:** 1857-01

**Authors:** 


*The Practical Anatomist; or, The Student's Guide in the Dissecting Room.* By J. M. ALLEN, M.D. Late Professor of Anatomy in the Medical Department of Pennsylvania College; Fellow of the College of Physicians, &c. With Two Hundred and Sixty-Six Illustrations. Philadelphia, Blanchard & Lea, 1856.

This is a well executed and substantially bound volume, of six hundred and thirty-one pages. From a hasty glance at its contents, we think it admirably arranged for rendering aid to the student in prosecuting dissections. Its illustrations are generally accurately drawn and highly useful. It will, doubtless, rank among the best *guides* in the study of practical anatomy that are now before the profession.

